# Hemorrhagic Shock Sensitized the Diaphragm to Ventilator-Induced Dysfunction through the Activation of IL-6/JAK/STAT Signaling-Mediated Autophagy in Rats

**DOI:** 10.1155/2019/3738409

**Published:** 2019-11-14

**Authors:** Li-Juan Zhang, Shao-Zhou Ni, Xian-Long Zhou, Yan Zhao

**Affiliations:** Emergency Center, Zhongnan Hospital of Wuhan University, 169 Donghu Road, Wuhan, Hubei 430071, China

## Abstract

Mechanical ventilation (MV) is a major life support technique for the management of trauma-associated hemorrhagic shock (HS). Ventilator-induced diaphragm dysfunction (VIDD), one of the most common complications of MV, has been well demonstrated in animal and human studies. However, few data are available concerning the effects of MV on diaphragm function in HS victims. In the present study, we found diaphragm muscle atrophy and weakness in HS but not in healthy animals after 4 hours of MV. The inhibition of autophagy resulted in reduced muscle fiber atrophy and improved forces. In addition, we observed diaphragmatic interleukin- (IL-) 6 overexpression and activation of its downstream signaling JAK/STAT in HS animals after MV, and either the neutralization of IL-6 or the inhibition of the JAK/STAT pathway attenuated autophagy, diaphragm atrophy, and weakness. Importantly, treatment with nonselective antioxidant exerted no protective effects against VIDD in HS animals. In addition, *in vitro* study showed that exogenous IL-6 was able to induce activation of JAK/STAT signaling and to increase autophagy in C2C12 cells. Moreover, the inhibition of JAK/STAT signaling abolished IL-6-induced cell autophagy. Together, our results suggested that HS sensitized the diaphragm to ventilator-induced atrophy and weakness through the activation of IL-6/JAK/STAT signaling-mediated autophagy in rats.

## 1. Introduction

Hemorrhagic shock (HS) is a severe outcome of traumatic injury. Patients who survive the initial phase of HS are at risk of multiple organ dysfunction syndrome (MODS), systemic inflammation, and oxidative stress [1]. It has been well demonstrated that HS can induce acute kidney injury (AKI) [2], liver injury [3], and acute lung injury (ALI) [4]. HS is also able to induce systemic inflammation, which is accompanied by increased levels of inflammatory cytokines such as interleukin- (IL-) 6, IL-8, and tumor necrosis factor-alpha (TNF-*α*) [5, 6]. In addition, it has been reported that oxidative stress was also involved in HS-induced MODS [7, 8]. A recent study reported that diaphragm function was preserved during HS in a rat model, and HS did not induce apparent inflammation in diaphragmatic tissues [9]. However, the sample collection and analysis were performed at a very early stage (within 2 hours) of HS in this study, and the subsequent events of HS have not been documented. Mechanical ventilation (MV), as a life support technique, has been widely used for traumatic HS patients at the first place after accident. MV use leads to diaphragm muscle atrophy and weakness, which has been linked to weaning difficulties and has been termed as ventilator-induced diaphragm dysfunction (VIDD) [10]. Although the mechanism of VIDD development has not yet been well explored, it is commonly accepted that inflammation and oxidative stress are the two major contributors [11, 12]. Since HS is able to induce systemic inflammation and oxidative stress, it is reasonable to speculate that HS possibly worsens diaphragm atrophy and weakness during MV. In the present study, we aim to test the hypothesis that short-time ventilation was able to induce diaphragm dysfunction in HS rats and to determine its underlying molecular mechanisms.

## 2. Methods and Materials

### 2.1. Animals

Adult male Wistar rats, weighting about 400 g, were purchased from the Charles River Laboratories (Beijing, China). All animals were kept in cages with a 12 : 12 light-dark cycle. Animal experiments were performed in accordance to the Guidelines of Animal Care and Use. This study was approved by the Animal Experiment Center of Zhongnan Hospital of Wuhan University (AUP-2018019). The water and food were provided *ad libitum*.

### 2.2. Grouping

Animal study: animals were randomly assigned into the following groups: (1) a control group (CON, *n* = 6): animals received cannulation and sham operation without either bleeding or MV; (2) a hemorrhagic shock (HS) group (*n* = 6): animals underwent HS (MAP maintained at 30-40 mmHg) for 60 min and then received resuscitation with shed blood and 0.9% saline to target a MAP of 80 mmHg for the next 4 hours; (3) a mechanical ventilation (MV) group (*n* = 6): animals received cannulation and a 4 h mechanical ventilation; (4) a HS+MV group (*n* = 6): animals underwent HS (MAP maintained at 30-40 mmHg) for 60 min and then received resuscitation with shed blood and 0.9% saline to target a MAP of 80 mmHg for the next 4 hours. At the same time, animals received MV for 4 hours; (5) a HS+MV+3-MA group (*n* = 6): HS animals received 4 hours of MV with the pretreatment of autophagy inhibitor 3-MA (i.p., 10 mg/kg); (6) a HS+MV+NAC group (*n* = 6): HS animals received 4 hours of MV with the treatment of N-acetylcysteine (NAC, i.p., 200 mg/kg); (7) a HS+MV+IL-6 monoclonal antibody (anti-IL-6 mAb) group (*n* = 6): HS animals received 4 hours of MV with the pretreatment of anti-IL-6 mAb (i.p., 5 mg/kg); and (8) a HS+MV+Rux group (*n* = 6): HS animals received 4 hours of MV with the treatment of JAK1/2 inhibitor ruxolitinib (i.p., 90 mg/kg). All drugs were given prior to the onset of MV. At indicated time points, blood samples were collected via the left femoral artery cannulation tube for blood gas analysis and blood cell counts. In addition, muscle tissues from the ventral part of the costal diaphragm were collected for biochemical and histological analysis, and a muscle strip about 1 cm from the ventral part of the costal diaphragm was purchased for the measurements of contractile properties.

### 2.3. Cell Study

Cells were seeded into four-well rectangular plates, the surface of which was coated with Matrigel (Becton, Dickinson and Co., Franklin Lakes, NJ, USA), at a density of 2.5 × 105 cells/well, with 3 mL of DMEM (25 mM glucose; Invitrogen, Carlsbad, CA, USA) supplemented with 10% FBS and 1% penicillin-streptomycin. The cells were maintained in an incubator at 37°C under a 5% CO_2_ atmosphere. Then, cells were cultured in the presence of IL-6 (30 *μ*g/mL) with or without JAK1/2 inhibitor Rux (20 *μ*M) at indicated concentrations. At various time points, cells were collected for Western blot and MitoSOX assay.

### 2.4. Reagents

Pentobarbital sodium was purchased from Amresco (Cleveland, OH, USA). Krebs-Henseleit Bicarbonate Buffer was purchased from M&C GENE Technology (Beijing) Ltd. (Beijing, China). Anti-IL-6 monoclonal antibody (anti-IL-6 mAb) for rats was purchased from InvivoGen (CA, USA). JAK1/2 inhibitor ruxolitinib (S1378) was purchased from Selleck Chemicals (Houston, TX). The primary antibodies including the anti-IL-6, anti-p-JAK1/2, anti-JAK1/2, anti-p-STAT3/5, anti-STAT3/5, and anti-LC3B-I/II were purchased form Abcam (Shanghai, China). MitoSOX™ Red Mitochondrial Superoxide Indicator was purchased from Invitrogen (CA, USA). Myosin Heavy Chain (NOQ7.5.4D) (ab11083), anti-Fast Myosin Skeletal Heavy Chain (MY-32) (ab51263), and anti-laminin were purchased from Abcam (Shanghai, China). Hydrogen Peroxide Assay Kit was purchased from BioVision (Palo Alto, CA, USA). Enzyme-linked immunosorbent assay (ELISA) kits for TNF-*α* (Cat no. RTA00), IL-1*β* (Cat no. RLB00), and IL-6 (Cat no. R6000B) were purchased from R&D Systems Inc. (Minneapolis, MN, USA). BCA protein assay kit was purchased from Beyotime (Shanghai, China). Lactate Assay Kit was purchased from BioVision (CA, USA).

### 2.5. Hemorrhagic Shock Model

A rat HS model was established as previously described [13]. Briefly, rats were anesthetized with pentobarbital sodium (i.p., 50 mg/kg; Amresco, Cleveland, OH, USA). Cannulation was performed using sterile polyethylene tubing on both the right and left femoral arteries. All the catheters and syringes were pretreated with heparinized normal saline (25 IU/mL, Shuanghe, Beijing, China). The right femoral artery cannulation tube was connected with a pressure transducer to a biological signal acquisition system for mean arterial pressure (MAP) monitoring (BL-420F, Chengdu Taineng Ltd., Chengdu, China). HS was performed by withdrawing blood over a 15 min interval via the left femoral artery cannulation tube. MAP was controlled at 30-40 mmHg for 60 min followed by resuscitation with shed blood and, if necessary, normal saline to target a MAP of 80 mmHg for the next experiment. The right jugular vein was cannulated for continuous infusion of normal saline (Baxter, Deerfield, IL) at a rate of 1 mL kg^−1^ h^−1^ and pentobarbital sodium (10 mg kg^−1^ h^−1^).

### 2.6. Mechanical Ventilation

Animals received MV for 4 hours since the end of resuscitation. The MV model was established as previously described [14]. In detail, animals were placed on a recirculating heating blanket and fixed under anesthesia. Then, animals were tracheostomized and connected to a volume-driven small animal ventilator (VentElite, Harvard Apparatus; Cambridge, MA, USA). The tidal volume (TV) was set at 5 mL/kg body weight, and the respiratory rate (RR) was set at 55-60 breaths/min. Breathing air was humidified and enriched with oxygen. To avoid ventilator-induced systemic hypoxia, the ventilator parameters and oxygen flow were adjusted to maintain PaO_2_ between 80 and 100 mmHg and PaCO_2_ between 35 and 45 mmHg during the entire study. Blood gas analysis was performed every two hours during the study.

### 2.7. Blood Sample Analysis

Arterial blood samples were collected every two hours for blood gas analysis during mechanical ventilation. Ventilator settings were adjusted according to blood gas results in order to maintain appropriate PaO_2_ and PaCO_2_. Blood gas analysis was performed using an i-STAT1 Analyzer (Abbott, Kyoto, Japan). Blood cell counts were determined prior to the onset of MV using an automatic blood cell analyzer Pentra MS CRP (HORIBA Medical, Kyoto, Japan).

### 2.8. MitoSOX Assay

Cellular mitochondrial ROS productions were measured using a MitoSOX Red Mitochondrial Superoxide Indicator according to the manufacturer's instructions. Briefly, MitoSOX Red Mitochondrial Superoxide Indicator (5 mM) was diluted with Hank's balanced salt solution (HBSS) buffer into a final concentration of 5 *μ*M (working solution). Cells were soaked on a cover glass with 1 to 2 mL of working solution and cultured for 10 minutes under 37°C. Cells were washed with prepared buffers three times. Staining and observation were conducted under a microscope.

### 2.9. Detection of Diaphragmatic H_2_O_2_ Production

Diaphragmatic H_2_O_2_ levels were detected using a commercial Hydrogen Peroxide Assay Kit according to the manufacturer's instruction. The absorbance was determined at 570 nm.

### 2.10. Evaluation of Cytokine Expression Levels

Tissue samples (100 mg) were homogenized with Triton-HEPES buffer (Sigma Aldrich, St. Louis, MO). Total protein was determined using a bicinchoninic acid (BCA) assay method (Beyotime, Shanghai, China). Diaphragmatic cytokines including TNF-*α*, IL-1*β*, and IL-6 and systemic IL-6 were measured by commercial ELISA kits according to the manufacturer's protocols.

### 2.11. Measurements of Diaphragmatic Lactate

Diaphragmatic lactate levels were determined using a commercial Lactate Assay Kit. Sample preparation, standard curve preparation, reaction, and calculation were performed according to the manufacturer's guidelines. Absorbance was determined at 450 nm. Lactate levels were presented as mmol/L.

### 2.12. Measurements of Muscle Contractile Properties

Each muscle strip was rapidly mounted in a tissue chamber containing Krebs-Henseleit (K-H) solution. The solution was bubbled with a gas mixture of 95% O_2_-5% CO_2_ and maintained at 27°C and pH 7.4. Muscle extremities were held in spring clips and attached to an electromagnetic force transducer. Muscle contractile properties were measured as previously described [15]. At the end of the experiment, each muscle cross-sectional area (CSA) (in cm^2^) was calculated from the ratio of muscle weight to muscle length at Lmax, assuming a muscle density of 1.06.

### 2.13. Immunofluorescence Staining

In the present study, immunofluorescence imaging was performed on paraffin-embedded tissues to evaluate the CSA of muscle fibers. The NOQ7.5.4D antibody was used for the identification of the slow-twitch fiber and the MY-32 antibody for the fast-twitch fiber. Muscle fibers are outlined by an antibody against laminin.

### 2.14. Western Blots

Western blots were performed in a standard protocol. Protein concentrations were determined using the BCA protein assay kit. Equal amounts of proteins were resolved by sodium dodecyl sulfate-polyacrylamide gel electrophoresis (SDS-PAGE), and the proteins were transferred to Hybond ECL membranes (Amersham, Buckinghamshire, UK). The membranes were incubated with primary antibodies including anti-JAK1/2, anti-p-JAK1/2, anti-STAT3/5, anti-p-STAT3/5, and anti-LC3B I/II at 4°C overnight. After washing with TBST, the membranes were probed with HRP-labeled secondary antibodies. The membranes were visualized using an enhanced chemiluminescence system (Kodak, Rochester, NY, USA). GAPDH was used as a loading control.

### 2.15. Statistical Analysis

Data are expressed as mean ± SD. Comparisons between groups were performed with one-way analysis of variance followed by post hoc tests for multiple comparisons. Association between diaphragm maximal tetanic forces and inflammatory cytokine levels was performed by Pearson correlation analysis. All statistical analyses were performed using GraphPad 5 (GraphPad Software, La Jolla, CA). A two-tailed *p* value less than 0.05 was considered significant.

## 3. Results

### 3.1. Short-Time Ventilation Induced Diaphragm Dysfunction in HS but Not in Healthy Animals through the Induction of Autophagy

**Figure 1:**
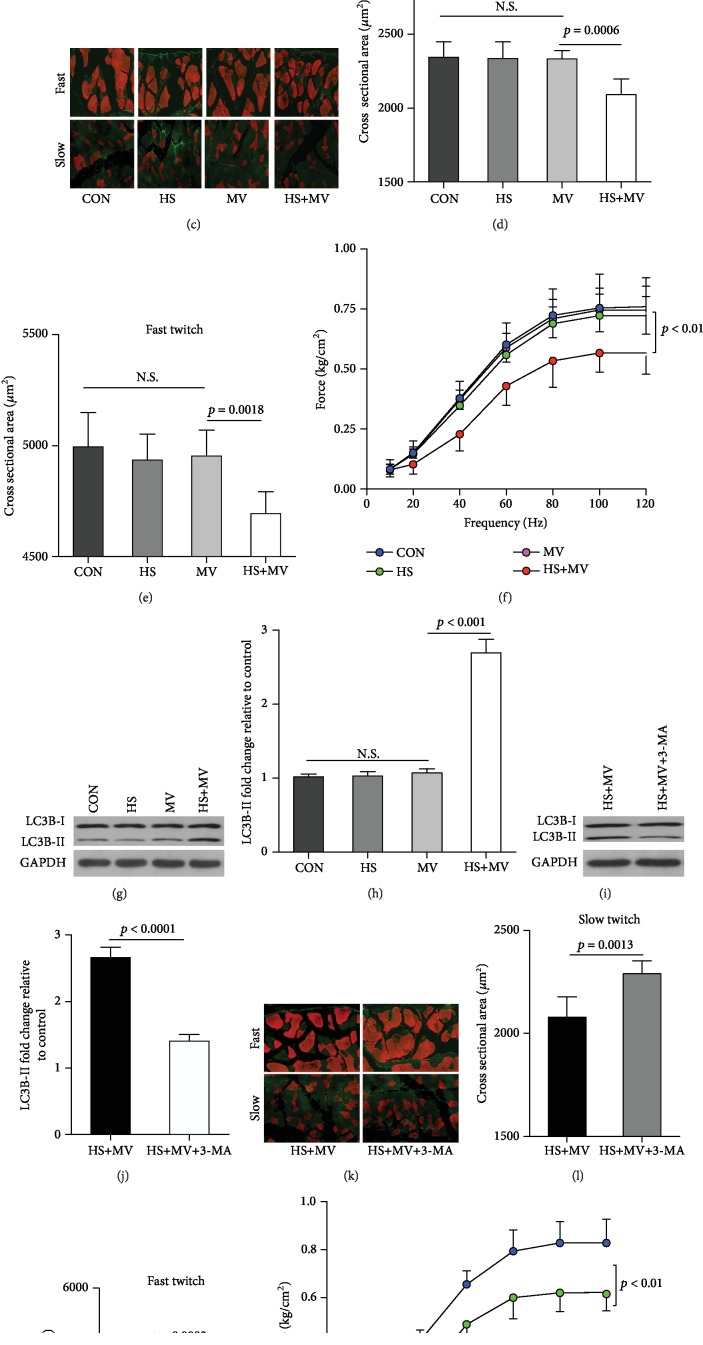
Short-time MV induced diaphragm dysfunction in HS animals (*n* = 6 in each group). (a) Diaphragm levels of lactate. (b) Hemoglobin levels. (c) Immunofluorescence staining for muscle fibers (40x). Both fast- and slow-twitch muscle fibers were stained as red, and the extracellular matrix were stained green. (d, e) Cross-sectional areas (CSA) of slow- and fast-twitch fibers. (f) Frequency-force curve. (g, h) Measurements of autophagic marker LC3B-I/II in MV and HS animals. (i, j) Measurements of LC3B-I/II in the diaphragm after 3-MA (an autophagy inhibitor) treatment. (k) Immunofluorescence staining for muscle fibers after 3-MA treatment (40x). Muscle fibers were stained red and extracellular matrices were stained green. (l, m) Cross-sectional areas (CSA) of slow- and fast-twitch fibers. (n) Frequency-force curve after 3-MA treatment. Measurements were repeated three times for each sample. MV = mechanical ventilation; HS = hemorrhagic shock.

The lactate levels of animals in the HS groups were significantly increased as compared to the non-HS groups (Figure 1(a)). After HS, the infusion of shed blood (12 ± 4 vs. 13 ± 5, *p* = 0.127) followed by normal saline (7 ± 5 vs. 6 ± 4, *p* = 0.314) was performed to achieve a target MAP of 80 mmHg in both the HS and HS+MV groups. In addition, blood cell counts demonstrated no significant differences in hemoglobin levels between groups prior to the onset of MV. Importantly, immunofluorescence staining demonstrated comparable muscle fiber sizes between the MV and control groups (Figure 1(c)). In addition, the cross-sectional areas (CSA) of either slow- or fast-twitch fibers were insignificantly decreased in the HS group as compared with the control group. These results indicated that short-term MV was unable to induce diaphragm atrophy in healthy rats. However, the CSA of slow- and fast-twitch fibers in the HS+MV group were significantly lower than those in the MV, HS, and control groups (Figures 1(d) and 1(e)). In addition, the frequency-force curve suggested that muscle forces were significantly decreased in the HS+MV group, but not in the MV and HS groups, as compared to the control group (Figure 1(f)). Since it has been widely accepted that the autophagy-lysosome pathway (ALP) contributes to the development of VIDD, we evaluated diaphragmatic autophagy in the present study. Western blots showed that the autophagic marker LC3B II/I ratio was markedly upregulated in the HS+MV group as compared to other groups (Figures 1(g) and 1(h)). In addition, inhibition of autophagy using 3-MA attenuated diaphragm atrophy and improved muscle forces (Figures 1(i)–1(n)). These results suggested that HS sensitized the diaphragm to VIDD in rats, and autophagy, at least, partly contributed to these pathological changes.

### 3.2. Increased IL-6 Expression but Not Oxidative Stress Participated in Short-Time Ventilation-Induced Autophagy and Diaphragm Dysfunction

**Figure 2:**
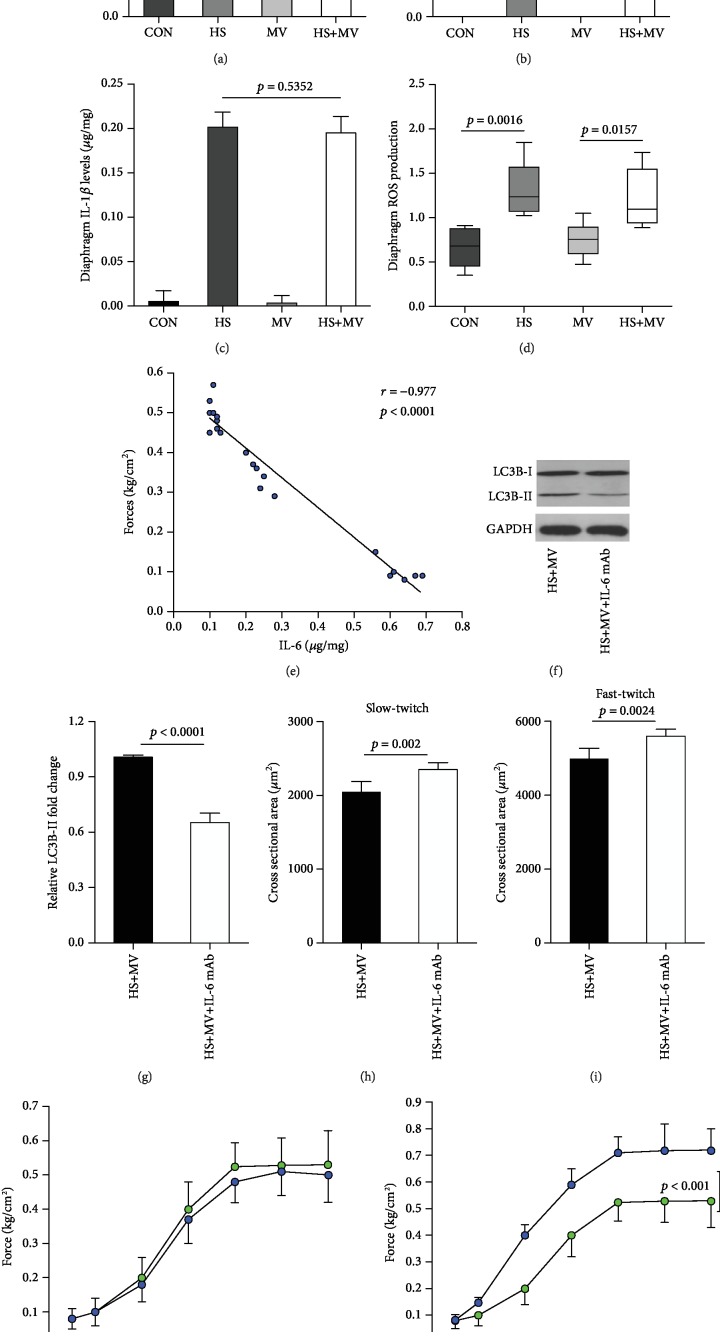
Short-time MV amplified IL-6 but not ROS induction in the rat diaphragm after HS (*n* = 6 in each group). Diaphragm expressions of (a) IL-6, (b) TNF-*α*, and (c) IL-1*β* were detected using ELISA kits. (d) Total ROS levels in the diaphragm. (e) Correlation analysis between maximal tetanic forces and IL-6 levels. (f, g) Assessment of autophagic marker LC3BI/II after IL-6 mAb treatment. (h, i) CSA of slow- and fast-twitch fibers after IL-6 mAb administration. (j, k) Measurement of muscle forces after antioxidant NAC and IL-6 mAb treatments. Measurements were repeated three times for each sample. MV = mechanical ventilation; HS = hemorrhagic shock.

It has been suggested that inflammation and oxidative stress are the two major contributors of VIDD. Therefore, we detected the expression of inflammatory cytokines and the production of reactive oxygen species (ROS) in the diaphragm after MV. Our results showed that IL-6 was significantly increased in the HS+MV group as compared to the other groups (Figure 2(a)). However, no significant differences in TNF-*α* and IL-1*β* expression levels were detected between groups (Figures 2(b) and 2(c)). Correlation analysis showed no significant association between diaphragmatic IL-6 and systemic IL-6 expressions (*r* = −0.208, *p* = 0.517). In addition, differences in total ROS levels were insignificant between the HS, MV, and HS+MV groups (Figure 2(d)). In addition, correlation analysis suggested that the expression levels of IL-6 were negatively associated with the maximal tetanic forces (Figure 2(e)). Moreover, the blockage of IL-6 using neutralizing IL-6 mAb resulted in decreased LC-3BII/I ratio with increased CSA of both slow- and fast-twitch fibers (Figures 2(f)–2(i)). Treatment with antioxidant NAC failed to attenuate diaphragm weakness (Figure 2(j)), whereas IL-6 mAb improved muscle forces (Figure 2(k)). These results suggested that IL-6 but not ROS is the major contributor to the onset of diaphragm atrophy and weakness at early stage in HS rats.

### 3.3. IL-6/JAK/STAT Signaling-Mediated Short-Time Ventilation Induced Autophagy-Associated Diaphragm Dysfunction

**Figure 3:**
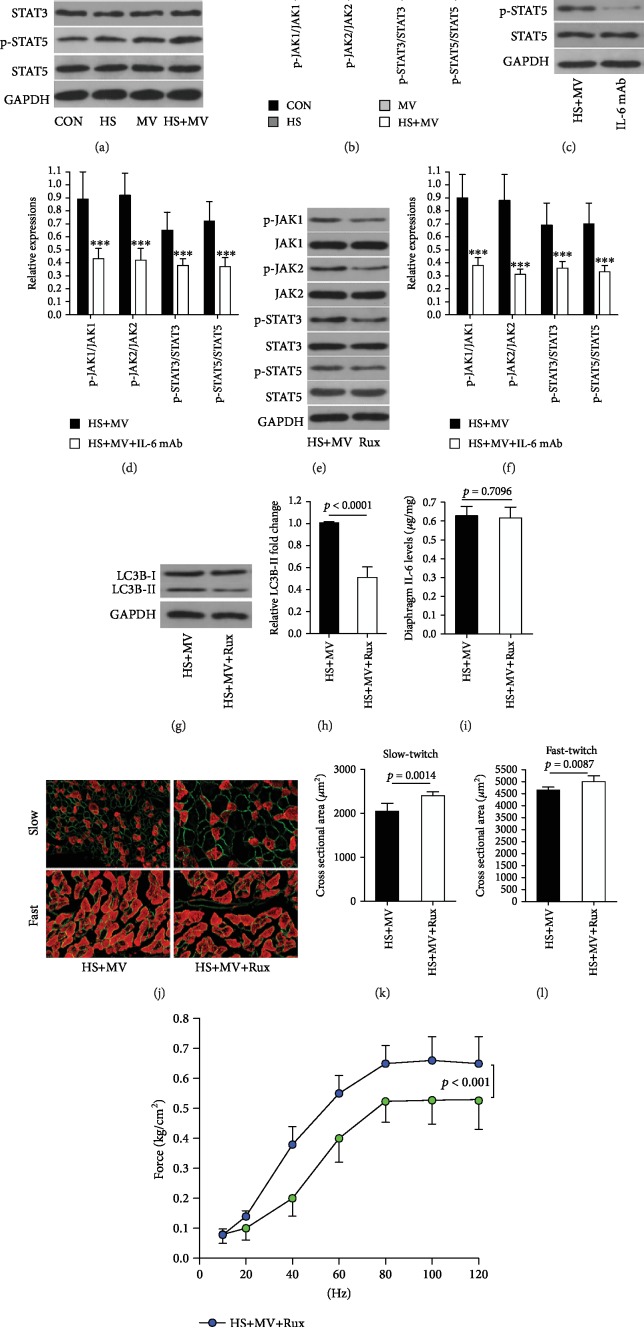
Roles of IL-6/JAK/STAT signaling in short-term MV-induced VIDD in HS animals (*n* = 6 in each group). (a, b) Western blots for JAK/STAT, the downstream signaling of IL-6, in the diaphragm after MV in HS animals. (c, d) Western blots for JAK/STAT signaling after IL-6 mAb administration. Western blots for (e, f) JAK/STAT signaling and (g, h) LC3B-I/II expression after JAK/STAT inhibitor ruxolitinib (Rux) treatment. (i) IL-6 expression in the diaphragm after Rux treatment. (j–m) Effects of Rux on diaphragm atrophy and weakness. Measurements were repeated three times for each sample. MV = mechanical ventilation; HS = hemorrhagic shock. ^∗∗∗^*p* < 0.001 vs. the HS+MV group.

**Figure 4:**
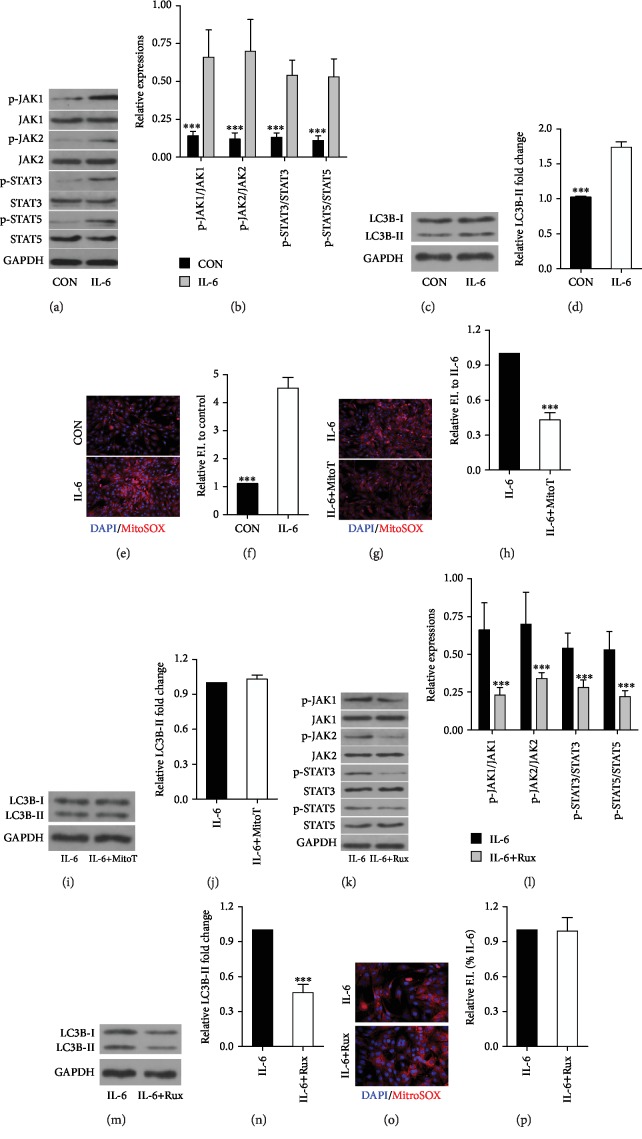
JAK/STAT signaling activation mediated the exogenous IL-6-induced autophagy *in vitro*. (a, b) Western blots for JAK/STAT signaling after IL-6 stimulation. (c, d) LC3B-I/II expression after IL-6 treatment. (e, f) Mitochondrial ROS production after IL-6 administration. (g, h) mitochondrial ROS production after mitochondria-targeted antioxidant MitoTEMPO (MitoT) treatment. (i, j) Western blots for LC3B-I/II expression after MitoT treatment. Western blots for (k, l) JAK/STAT signaling and (m, n) LC3B-I/II expression after JAK/STAT inhibitor ruxolitinib (Rux) treatment. (o, p) Mitochondrial ROS production after Rux treatment. Measurements were repeated three times for each sample. ^∗∗∗^*p* < 0.001 vs. the IL-6 group.

As described above, the blockage of IL-6 is able to protect HS animals against VIDD after a short time of MV. Here, we aim to investigate the underlying molecular mechanism. The signal transduction of IL-6 involves the activation of JAK, then leads to the activation of transcription factor STAT. Our results found that MV induced JAK/STAT signaling activation in the diaphragm of HS animals (Figures 3(a) and 3(b)), and the blockage of IL-6 using neutralizing IL-6 mAb downregulated the activation of JAK/STAT signaling (Figures 3(c) and 3(d)). In addition, JAK/STAT inhibition resulted in decreased LC3B II/I ratio in the diaphragm with attenuated atrophy and increased forces. However, JAK/STAT inhibition did not alter diaphragmatic IL-6 expressions (Figures 3(e)–3(m)). In addition, cell study showed that IL-6 was able to induce activation of JAK/STAT signaling (Figures 4(a) and 4(b)) in C2C12 cells with upregulated LC3B-II/I ratio and increased mitochondrial ROS production (Figures 4(c)–4(f)). However, mitochondria-targeted antioxidant MitoTEMPO treatments failed to downregulate LC3B-II/I ratio (Figures 4(g)–4(j)). Moreover, JAK/STAT signaling inhibition also resulted in decreased LC3B-II/I ratio without alterations in mitochondrial ROS expression (Figures 4(k)–4(p)). These results suggested IL-6/JAK/STAT signaling activation was able to induce autophagy formation *in vitro*.

## 4. Discussion

The major findings of this study can be summarized as follows: (1) a 4 h MV failed to induce diaphragm muscle atrophy and weakness in healthy animals. However, HS animals with effective resuscitation showed detectable diaphragm muscle atrophy and weakness after a short period (4 h) of MV; (2) autophagy contributed to diaphragm atrophy and weakness in HS animals that underwent 4 hours of MV; and (3) short-time ventilation induced expression of IL-6 in the diaphragm without apparent oxidative stress in HS animals, and the blockage of IL-6/JAK/STAT signaling attenuated autophagy-mediated diaphragm dysfunction in HS animals.

It has been reported that diaphragmatic inactivity promotes an increase in mitochondrial ROS emission, mitochondrial oxidative damage, and mitochondrial respiratory dysfunction [16]. The excessive ROS production and oxidative stress accelerate the protein degradation and inhibit protein synthesis [17], which finally resulted in muscle fiber atrophy and weakness. Therefore, the mitochondrial oxidative stress has been considered a key upstream regulator in the development of VIDD [18]. It has been demonstrated that visible diaphragm dysfunction can be induced by controlled mechanical ventilation (CMV) over 6 hours in healthy animals [19]. In the present study, animals received MV for only 4 hours, which was generally unable to induce diaphragm dysfunction in healthy animals. However, we observed apparent diaphragm atrophy and weakness in animals of the HS+MV group but not in the HS and MV groups. These results indicated that the diaphragm was sensitized to ventilator-induced atrophy and weakness in HS animals. In addition, instead of ROS production, the overexpression of IL-6 is likely the major contributor to the rapid development of VIDD in HS animals.

Autophagy is a catabolic process characterized by the formation of specialized vesicles that engulf cytoplasmic elements and then fuse with lysosomes to degrade their contents [20]. Numerous studies have confirmed that prolonged MV is able to induce diaphragmatic autophagy in both animal models [21] and in humans [19]. However, the role of autophagy in VIDD is controversial based on current evidences. Hussain and colleagues demonstrated that the activation of autophagy during VIDD provides a protective role [19]. However, a very recent study revealed that autophagy is required for VIDD and that autophagy inhibition reduces MV-induced diaphragm ROS production and proteolysis [22]. These experiments were performed on healthy animals with MV for 6 or 12 hours. In the present study, we observed diaphragmatic autophagy in HS animals but not in healthy animals after 4 hours of MV. In this study, we established a HS+MV model to investigate the roles of autophagy in diaphragm atrophy and weakness. All animals in the HS+MV group underwent HS and reperfusion prior to the onset of MV. Therefore, ischemic/reperfusion injury (IRI) probably occurred in the major organs including the diaphragm. Previous studies have demonstrated that autophagy is probably a protective mechanism during IRI [23, 24]. Importantly, Azuelos and colleagues found that autophagy is induced by MV but is not responsible for diaphragmatic weakness. The authors even proposed that autophagy may instead be a beneficial adaptive response that can potentially be exploited for therapy of VIDD [21]. In the present study, our results showed that the inhibition of autophagy resulted in decreased protein degradation, reduced muscle atrophy, and improved forces. These results first suggested that autophagy contributes to the development of diaphragm atrophy and weakness in HS animals following a short period of MV.

Previous studies have demonstrated that either oxidative stress or inflammatory cytokines are able to induce autophagy in skeletal muscles [25, 26]. In this study, we observed significant increases in IL-6 levels in the diaphragm in the HS+MV group. JAK/STAT signaling is a major downstream signaling pathway of IL-6 and its receptor, which has been proven to be critical in VIDD [27]. Our results showed that the induction of autophagy in the present animal model was dependent on the activation of IL-6/JAK/STAT signaling. However, STAT3 has been demonstrated as a new autophagy regulator, which interacts with the PKR kinase to reduce autophagic pathways [28]. Very recently, Petrof and colleagues reported that increased IL-6 expression in the diaphragm during MV plays an important role in pathogenesis of the early diaphragm weakness [29]. In the present study, the activation of STAT3/5 was observed in the diaphragm. Importantly, the inhibition of JAK activation with ruxolitinib resulted in inactivation of STAT3/5 and decreased cell autophagy. Previous study demonstrated that the inhibition of JAK/STAT prevents oxidative stress-induced protein oxidation and polyubiquitination and recovers mitochondrial function in cultured muscle cells [27]. However, our study showed that the inhibition of JAK/STAT signaling was unable to diminish IL-6-induced mitochondrial ROS production in skeletal muscle cells. In addition, administration of nonselective antioxidant NAC failed to attenuate diaphragm atrophy and weakness; however, the blockage of IL-6/JAK/STAT signaling resulted in improved diaphragm function. Together, these results suggested that the molecular changes of diaphragm muscle atrophy and weakness in the HS+MV model differs from that in the prolonged MV model. Collectively, our data indicated that HS sensitized the diaphragm to ventilator-induced diaphragm atrophy and weakness through the activation of IL-6/JAK/STAT signaling-mediated autophagy.

There are two major limitations in the present study. First, our results demonstrated that ROS production/oxidative stress is not a major contributor to the early pathogenesis of VIDD in HS rats. However, only a single dose of nonselective antioxidant NAC has been used *in vivo*; more evidences are required to elucidate the roles of ROS production/oxidative stress in short-term MV-induced diaphragm atrophy and weakness after HS. Second, our results suggested that short-term MV induced diaphragm dysfunction in HS animals after resuscitation but not in healthy animals. In clinical practice, critically ill patients are commonly ventilated for more than 12 hours, and VIDD is reported in up to 60%-80% among those patients [30]. Therefore, whether prolonged MV (≥12 hours) induces more severe diaphragm weakness and atrophy in HS animals than in non-HS animals remains unclear. Further studies are required to determine the effects of HS on diaphragm function in animals receiving prolonged MV.

## Data Availability

The data used to support the findings of this study are available from the corresponding author upon reasonable request.
